# An evaluation of the predictive validity of the URICA and ANSOCQ scales for weight gain in adults with AN in an outpatient eating disorders program: a prospective cohort study

**DOI:** 10.1186/s40337-017-0180-0

**Published:** 2017-11-13

**Authors:** Jessica Green, Andrea Phillipou, David Castle, Leonardo Cistullo, Richard Newton

**Affiliations:** 1 Austin Hospital, Heidelberg, Australia; 2 Swinburne University, Hawthorn, Australia; 3 St Vincent’s Health, East Melbourne, Australia; 4 University of Melbourne, Parkville, Australia; 5 Monash University, Clayton, Australia; 6 Peninsula Health, Frankston, Australia

**Keywords:** Anorexia nervosa, Stage of change, University of Rhode Island Change Assessment Scale, Anorexia nervosa stage of change questionnaire, Transtheoretical model

## Abstract

**Background:**

The Transtheoretical Model (TTM) which focuses on stage of change has been the main conceptual model used in understanding the lack of motivation to change in patients with Anorexia Nervosa (AN). Whilst there is evidence to support the prognostic value of the TTM in AN, this evidence base sufferers from limitations including limited studies in adults and none in outpatient populations. The primary aim of this study was to clarify whether readiness to change, as measured by the University Rhode Island Change Assessment Scale (URICA) and the Anorexia Nervosa Stages of Change Questionnaire (ANSOCQ) could predict weight gain in adults with AN following treatment in an outpatient setting.

**Methods:**

This was a prospective cohort analysis, which selectively used data from an existing clinical database at an outpatient eating disorders service. 119 patients met eligibility criteria and were included in this study. This included all adult patients who had a diagnosis of AN and were assessed, but not necessarily treated at the outpatient eating disorders program (Group 1). A subgroup of 63 patients (Group 2) was also analysed which only included patients who had received treatment at the program. Baseline measures included the URICA score, the ANSOCQ score, the Eating Disorders Examination Questionnaire (EDE-Q) and body mass index (BMI). BMI was also measured on discharge.

**Results:**

The URICA scale had poor predictive validity for weight gain (*r* = 0.05, *p* = 0.725). The ANSOCQ had moderate predictive validity (Pearson’s *r* = 0.57, *p* = 0.007), and accounted for 32.7% of variance in weight gain. The URICA and ANSOCQ were moderately correlated in both groups. The URICA was moderately predictive of symptom severity, measured by the EDE-Q in both groups. The ANSOCQ was moderately correlated with the EDE-Q scores in both Groups 1 and 2.

**Conclusions:**

To the authors’ knowledge this is the only study evaluating stage of change, in an adult outpatient population with AN. The findings of this study suggest that while both the URICA and ANSOCQ were associated with eating disorder symptom severity, only the ANSOCQ was able to predict weight gain in outpatients with AN suggesting its greater utility in this context.

## Plain English summary

Assessment of stage of change often forms a core part of understanding and treating Anorexia Nervosa (AN). There is a body of evidence which suggests that stage of change is a valuable tool for predicting treatment outcomes. Yet this body of evidence is limited in adults with AN, and to the authors’ knowledge, there is no evidence in outpatient populations with AN. The main aim of this study was to clarify whether stage of change could be used to predict treatment success in adult outpatients with AN, as measured by weight gain in patients with AN following treatment in an eating disorders outpatient program. Stage of change was measured with two questionnaires: the University of Rhode Island Change Assessment Scale (URICA) and the Anorexia Nervosa Stages of Change Questionnaire (ANSOCQ). While both the URICA and ANSOCQ were associated with eating disorder symptom severity, only the ANSOCQ was able to predict weight gain in outpatients with AN suggesting its greater utility in this context.. This has implications for the assessment of AN.

## Background

A core issue relating to the psychopathology of Anorexia Nervosa (AN) is limited insight into the disordered pattern of eating and weight as being problematic, which is associated with a lack of motivation for recovery and change [1–3]. The Transtheoretical Model (TTM), has been the main conceptual model used in understanding the lack of motivation in patients with AN [4]. The TTM uses the Prochaska and DiClemente Stages of behavioural change and suggests targeted treatments depending on stage (ie. precontemplation, contemplation, preparation, action and maintenance) [5]. The core constructs of this model include stages of change, self-efficacy and decisional balance and several cognitive and behavioural processes of change [6]. This model has been met with mixed views. Wilson and Schlam [7] have criticised the model for its categorical nature, suggesting that motivation to change might be better assessed using a continuum as opposed to distinct categories. They also note that “the stages do not constitute discrete categories because it is possible for individuals to be in more than one stage at the same time” [7], Yet, as Hoetzel et al. [8] note, “to date, the research literature does not provide any alternative solution to this categorical model and the categorical assessment approach” [8].

Despite these concerns regarding the TTM, the current body of evidence lends support to the prognostic value of this model in the context of eating disorders. Dray and Wade [9] conducted a broad and extensive literature review which evaluated the value of motivational interviewing and the TTM in the treatment of eating disorders. They concluded that motivational interviewing has poor evidence for efficacy in the treatment of eating disorders, but the TTM has sufficient evidence to suggest that initial stage of change is predictive of a variety treatment outcomes in eating disorders [9]. Clausen et al. [10] built on the work of Dray and Wade, and conducted a systematic review evaluating the predictive value of instruments which assess stage of change in eating disorders [10]. They identified five studies which evaluated the prognostic value of pre-treatment stage of change in AN. Of the five studies identified, only two evaluated an adult population [11, 12]whilst the other three evaluated an adolescent population [13–15]. Clausen et al. [10] concluded that pre-treatment stage of change is predictive of post-treatment outcomes. However, the limitations of this evidence were also noted by the authors: a relatively small body of research, issues of heterogeneity regarding measures used to assess motivation, the type and length of intervention applied, variable follow up times, and many studies with low statistical power due to a small sample size. It must also be noted that there were no studies identified which evaluated this question in an outpatient population with AN.

This study therefore aimed to fill a gap in the research by evaluating the prognostic value of stage of change in adults with AN in an outpatient setting. To the authors’ knowledge, there are no previous studies which have evaluated this clinical question, and only minimal studies evaluating stage of change in AN. Two assessment tools were used to assess stage of change: the University of Rhode Island Change Assessment Scale (URICA) and the AN Stage of Change Questionnaire (ANSOCQ). Given the limitations noted by Wilson and Schlam [7], it was decided that the global readiness to change score, as calculated by each of these measures respectively, rather than specific stage of change, should be used to assess pre-treatment stage of change.

The URICA Scale is the most widely used dimensional measure for assessing stage of change [16, 17]. This scale was initially developed for patients entering addiction treatment [18, 19]. It consists of a 32-item self-rated scale which aims to evaluate readiness to change. Each item is rated out of five by the participant using a likert-scale, in which a score of one equates to “strongly disagree” and five equates to “strongly agree”. Using a scoring grid, sub-scores are given for each of the Prochaska and DiClemente stages of behavioural change (ie. precontemplation, contemplation, action and maintenance). An overall readiness to change score is calculated using a weighted average of the four sub-scores, where 14 is the maximum possible score. A higher score corresponds to increased readiness to change.

The URICA Scale is considered to have robust psychometric properties [20, 21]. However, it has not been validated in an eating disorder context. The scale was intended to assess stage of change in any disorder, thus the statements and terms used in the scale are generic in nature, such as “problems that need changing” or the need for “self-improvement”, Rieger et al. [22] make the argument that these phrases may be misinterpreted by the participant to refer to a number of issues that may not be in any way related to AN, such as relationship problems or emotional issues. This could understandably skew the results of the scale, making it difficult to interpret with respect to AN. For example, the statement “I have been thinking that I might want to change something about myself” may be interpreted as being related to any number of issues which may not be related to the patient’s AN diagnosis, and if the individual strongly endorsed this statement on that basis, their score would be overestimated. [22] The authors of this study therefore hypothesised that the URICA Scale would be of limited value in assessing stage of change in AN.

The ANSOCQ was developed by Rieger, et al. [22] to fill a perceived gap in assessment tools available for the assessment of readiness to change in patients with AN. The scale has been validated by the authors for use in this population, with good predictive value for weight gain. It is also thought to have robust psychometric properties [10, 22, 23]. The scale consists of a 20-item self-rated scale. An overall score out of 100 is given, with higher scores corresponding with increased motivation to change [22, 23].For each item, the participant endorses one or more of five statements, which is then scored out of five. Each statement corresponds with a stage of change, and is scored appropriately (ie. precontemplation scores one point, contemplation scores two points, and so on). Participants may endorse more than one statement, and if this is the case, the two statements are averaged out to produce a single score for the item. An overall stage classification score can be obtained by dividing the total score by the number of items (ie. 20). Each of the items also evaluates one of the core features of AN symptomology. Thus it is specifically designed to assess stage of change in AN. The statements themselves are focused, leaving them less open to interpretation than the URICA scale [22].

The URICA has not been extensively evaluated in the eating disorders context. Von Bratchel, et al. [24] evaluated the predictive validity of the short version of the URICA Scale (URICA-S) for drop-out rates in 179 women with an eating disorder receiving an internet-based intervention. It was found that the URICA-S was not predictive of drop-out from treatment [24]. Mander et al. [25] studied an inpatient population with AN. The URICA-S was used in 39 patients throughout early, middle and late stages of inpatient treatment. The study found that the URICA-S was not predictive of change in Body Mass Index (BMI) [25].

Rieger et al. [22] evaluated both the ANSOCQ and URICA in an adult inpatient population (*n* = 71) with AN. The study investigated predictive validity of both of these tools in predicting weight gain in adult patients with AN and found that the ANSOCQ had a statistically significant predictive validity (*t* = 1.99, *p* = 0.05), whilst the URICA did not (*p* = 0.09).

Rieger et al. [22, 23] also investigated the correlations between the URICA and the ANSOCQ, as both of these scales aim to assess motivation to change. It would therefore be expected that the scores would correlate. Indeed, they found a statistically significant correlation between the ANSOCQ and URICA across all four of the stages of change subscales; −0.64 (*p* = ..000) for the precontemplation scale, 0.66 (*p* < 0.001) for the contemplation scale, 0.72 (*p* = 0.000) for the action scale and 0.34 (*p* = 0.004 for the maintenance scale).

The rationale for this study is as follows. The TTM is the main conceptual model used in understanding lack of motivation in eating disorders. Yet, as has been noted above, there have also been criticisms of the categorical nature of the model and debate over how to best measure stage of change. Determination of the most accurate and robust tool for assessing stage of change with respect to AN is crucial for a number of reasons. Firstly, the tool can be used to inform future research, leading to more accurate and consistent research methodologies. Secondly, the tool can be used to valuably aid clinical practice and assessment.

The ability to predict treatment outcomes in an AN population is of great value clinically. Prognostic information is of importance to clinicians in anticipating and preparing for the likely course of treatment. Prognosis is also important to patients and their families as it can be used to inform psychoeducation and appropriate and realistic expectations of treatment outcomes.

### Study aims

The primary aim of this study was to assess the predictive validity of both the URICA scale and the ANSOCQ in a novel population (ie. adult outpatients with AN). This was measured by evaluating the predictive validity of the URICA scores and ANSOCQ scores for predicting weight gain in patients with AN following treatment in an outpatient eating disorders program.

Secondary aims of this study included correlating the URICA scores with ANSOCQ scores, and evaluating whether the URICA and ANSOCQ scores were correlated with symptom severity, as measured by the Eating Disorder Examination Questionnaire (EDE-Q) in patients with AN.

To the authors’ knowledge, this is the only study which has evaluated these questions in an outpatient population with AN. Furthermore, this study adds to a limited body of research, which has suffered from heterogeneity in methodologies, and with several studies having low sample sizes, and thus being poorly powered to demonstrate their outcomes. This study uses a clinical sample which the authors believe should therefore be generalizable to a clinical outpatient population and be well powered to demonstrate study outcomes.

## Methods

This was a prospective cohort analysis, which selectively used data from an existing clinical database at an outpatient eating disorders service.

### Outline of body image eating disorders treatment and recovery service

The study was based out of the Body Image Eating Disorders Treatment and Recovery Service (BETRS), which is an outpatient program based out of two metropolitan tertiary health services in Melbourne, Australia. Patients are generally referred to the program by primary and secondary care providers for the treatment of eating disorders.

All patients referred to BETRS receive an initial assessment. After assessment, patients are allocated to treatment at BETRS, referred to external treatment providers or treated as inpatients at a specialised inpatient eating disorders unit. 47% of patients assessed at BETRS are considered appropriate for referral to external services and receive no further treatment at BETRS. These patients usually have lower illness severity, and are considered appropriate for treatment by other community services.

### Recruitment

Researchers recruited all patients assessed at BETRS between October 2010 and December 2016. 470 patients were included over this time.

### Consent

Participants were asked to provide written consent for use of their data in the BETRS database to be used for any future studies conducted by BETRS. 302 patients provided consent, and 168 participants did not and their data has been excluded from this specific study. It is important to note that participants who did not consent appear to have coincided with administrative challenges in data collection and staffing deficits, and may not be due to participants declining to participate.

### Ethics

Ethics approval was provided by St Vincent’s Human Ethics Research Committee for the collection of patient data in the BETRS database, and use of this data in subsequent studies.

### Data collection

At their initial assessment at BETRS, research staff provided all patients with an assessment pack containing various self-report outcome measures, including demographic data, EDE-Q, ANSOCQ and URICA. Initial diagnosis was made by BETRS clinicians using the Mini International Neuropsychiatric Interview [26]. The diagnosis was later confirmed with a consultant psychiatrist at the team meeting, who took into account the Diagnostic Statistical Manual (DSM) IV-TR criteria, and from 2013, the DSM-5 criteria for AN [27, 28]. The authors have previously published two papers evaluating the effect of the change in the diagnostic criteria from DSM IV-TR to DSM-5, based on this same cohort. The analysis within this paper included some data that would have previously been excluded but the authors do not believe that the impact on the results is skewed in any way by the additional sample [29, 30]. The following information was collected from the patient’s file, where available: primary diagnosis, AN subtype, Body Mass Index (BMI) at assessment and discharge, outcome of BETRS assessment and treatment received. BMI was calculated based on height and weight which were measured by BETRS clinicians using a single set of scales and stadiometer.

### Outcome measures

The ANSOCQ and URICA scales and scoring information are described in the study introduction. These scales were used to measure stage of change in this study. In interpreting the URICA scores according to the four sub-scales, a score of 8 or lower is classified as Precontemplation, a score of 8–11 is classified as Contemplation, a score of 11–14 is classified as Preparation or Action [31, 32].

In interpreting the ANSOCQ scores, the following average scores correspond to the various stages of change: A score of less than 1.5 is classified as Precontemplation, a score of 1.5–2.4 is classified as Contemplation, a score of 2.5–3.4 is classified as Preparation, and a score of over 4.5 is classified as Maintenance. [22, 23].

The EDE-Q is a 28-item self-report questionnaire, and is generally viewed as a gold standard measure of eating disorder psychopathology. It evaluates the range and severity of eating disorder features and generates scores across four subscales including weight concern, shape concern, eating concern and restraint [33]. Each subscale is scored out of six. A global score is calculated by calculating a mean for the four subscale scores, thus the maximum possible global score is six. Population norms are also available for the EDE-Q [34]. Higher scores indicate greater levels of symptom severity.

Change in BMI was a primary outcome measure in this study, and has been interpreted as a marker of success for patients with AN following treatment at BETRS.

### Inclusion and exclusion criteria

**Fig. 1:**
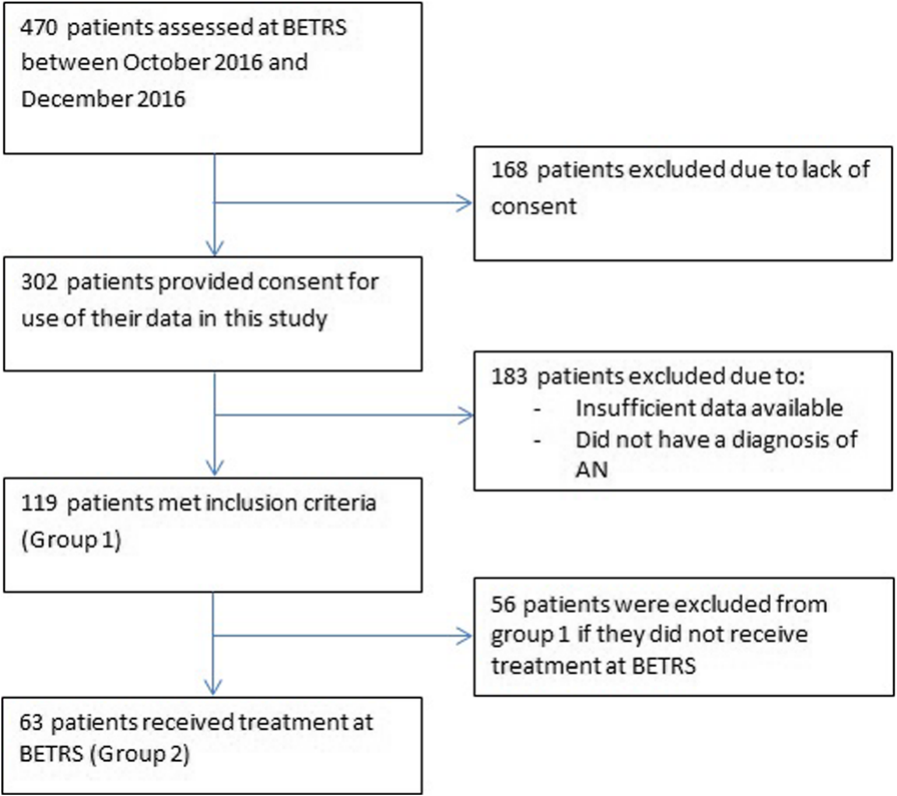
Patient inclusion flowchart

Participants were included in the study if the following criteria were met: first episode of care at BETRS only was included in the study (ie. subsequent episodes of care for the same patient were not included); and they had a diagnosis of AN. Participants were excluded from the study if they failed to provide adequate consent and/or minimum data was unavailable for a given analysis. Data was checked for normality, and outliers were removed based on box plots of the data. Patient inclusion and exclusion data is represented below in Fig. 1.

### Patient groups

Two patient groups were considered in this study. Group 1 consisted of all patients who were assessed at BETRS (*n* = 119). Group 2 consisted of a sub-group of these patients who received assessment *and* treatment at BETRS (*n* = 63). Baseline data and discharge data was available only for Group 2. Group 2 was therefore the cohort of interest with respect to the primary study aim. Group 1 was also included in study analyses for baseline measures as the larger cohort could add statistical power to baseline analyses.

### Statistical analysis:

IBM SPSS version 24 was used for the statistical analysis. Linear regression analysis (forced entry) was used, with alpha set at .05 for all analyses. Five models were generated. To assess the predictive validity of the URICA score on weight gain, the URICA score was included as the independent variable (IV) and weight gain from assessment to discharge as the dependent variable (DV). To assess the predictive validity of the ANSOCQ score on weight gain, the ANSOCQ score was included as the IV and weight gain from assessment to discharge as the DV. To assess the correlation of the URICA and EDE-Q score, the URICA score was included as the IV and the EDE-Q was the DV. To assess the correlation of the ANSOCQ and EDE-Q score, the ANSOCQ score was included as the IV and the EDE-Q was the DV. Finally, to assess correlation between the ANSOCQ and URICA, the URICA was included as the IV and the ANSOCQ as the DV.

Correlational analyses were undertaken with Pearson’s correlations. Outliers were identified with box plots (i.e. values falling 1.5 times the interquartile range above the third quartile or below the first quartile) and removed from analyses.

## Results

### Patient demographics

302 patients gave consent for their data to be used in this study and 119 of these patient episodes met study inclusion criteria. The mean time in treatment was 0.32 years (SD = 0.50).

Patients were analysed in two groups. Group 1 (*n* = 119) included all patients who were assessed at BETRS meeting the inclusion criteria. This data is represented above in Fig. 1. The mean age of this group at baseline was 28.34 (SD = 9.63). Demographic data for this patient group can be viewed in Table 1. Baseline outcome measures are presented in Table 2.

**Table 1:** Demographic data for Group 1

		n (*N* = 119)	%
Gender	Male	5	4.2
	Female	114	95.8
Employment	Student	39	32.8
	Employed Full Time	12	10.1
	Employed Part Time	24	20.2
	Home Duties	3	2.5
	Unemployed	12	10.1
	Unable to Work Because of Illness	19	16.0
	Other	5	4.2
	Data unavailable	5	4.2
Marital status	Never Married	78	65.5
	Widowed	1	.8
	Divorced	3	2.5
	Separated	4	3.4
	Married (including de facto)	18	15.1
	Data unavailable	15	12.6
AN subtype	Restrained Eating	81	68.1
	Purging	7	5.9
	Data unavailable	31	26.1

**Table 2:** Baseline outcome measure scores for Group 1

	Mean Score	SD
EDE-Q	3.92^a^	1.64
URICA	8.96^b,c^	2.39
ANSOCQ	2.67^d,e^	0.58

^a^Data was available for 88 participants

^b^A mean URICA score of 8.96 corresponds to the “contemplative” stage of change [31, 32]

^c^Data was available for 95 participants

^d^A mean ANSOCQ score of 2.67 corresponds to the “preparation” stage of change [22]

^e^Data was available for 37 participants

The demographic data for Group 2 is presented in Table 3. The mean age of this group was 29.03 (SD = 9.79). The mean BMI at assessment for this group was 15.72 (SD = 2.39). The mean BMI on discharge was 16.43 (SD = 2.47). Baseline outcome measures for Group 2 are presented in Table 4.

**Table 3:** Demographic data for Group 2

		n (N = 63)	%
Gender	Female	60	95.2
	Male	3	4.8
Marital status	Never married	37	58.7
	Divorced	2	3.2
	Separated	3	4.8
	Married (including de facto)	12	19.0
	Not stated	9	14.3
Highest level of education attained	Secondary Years 7–10	2	3.2
	Secondary Years 11–12	12	19.0
	Tertiary Commenced	21	33.3
	Tertiary Completed	19	30.2
	Vocational	2	3.2
	Not Stated/Inadequately Described	2	3.2
	Other	2	3.2
	Unknown	3	4.8
AN subtype	Restrained Eating	41	65.1
	Purging	3	4.8
	Not assessed/data unavailable	19	30.2
Treatment received	Austin Eating Disorder inpatient unit	17	27.0
	Day patient program	33	52.4
	Family therapy	1	1.6
	Group program	2	3.2
	Individual outpatient follow up	10	15.9

**Table 4:** Baseline outcome measures for Group 2

	Mean Score	SD
EDE-Q	3.76^a^	1.68
URICA	9.29^b^	2.30
ANSOCQ	2.65^c^	0.58

^a^Data available for 43 participants

^b^Data available for 52 participants

^c^Data available for 25 participants

### Predictive validity of the URICA scores for weight gain in AN following treatment

The URICA scores were correlated against change in weight from admission to discharge in patients with AN in Group 2 only, due to unavailability of discharge data for Group 1 (as these individuals were not treated at BETRS). 46 patient episodes were analysed due to data availability, and one outlier was excluded. The URICA scores were not significantly correlated with weight gain following treatment (Pearson’s *r* = 0.05, *p* = 0.725).

### Predictive validity of the ANSOCQ scores for weight gain in AN following treatment

Similarly to the analyses for the URICA scores, the ANSOCQ scores were correlated against change in weight from admission to discharge in patients with Anorexia nervosa in Group 2 only. 21 patient episodes were analysed due to data availability, and no outliers were excluded. The ANSOCQ was found to have a significant positive predictive value of moderate strength for weight gain following treatment at BETRS (Pearson’s *r* = 0.57, *p* = 0.007). The ANSOCQ scores accounted for 32.7% of the variance in weight gain.

### Correlations between the URICA and ANSOCQ scores

The URICA and ANSOCQ scores were correlated in both Groups 1 and 2. In Group 1, 30 patient episodes were analysed, as data was available for 31 patient episodes, and one outlier was excluded. A significant positive correlation of moderate strength was found between the URICA and ANSOCQ scores (Pearson’s *r* = 0.47, *p* = 0.009). A linear regression analysis was performed with the URICA scores as a predictor of variance of the ANSOCQ scores. The URICA scores were found to explain 22% of the variance in the ANSOCQ scores. In Group 2, 21 patient episodes were analysed, as data was available for 23 patients, and two outliers were removed. A significant positive correlation was again found between the URICA and ANSOCQ scores (Pearson’s *r* = 0.46, *p* = 0.031), with the URICA scores found to explain 21% of the variance in the ANSOCQ scores.

### Correlations between the URICA scores and symptom severity

The URICA and EDE-Q scores were correlated in Groups 1 and 2. In Group 1, 68 patient episodes were analysed, as data was available for 69 episodes, with one outlier removed. A significant negative correlation of weak strength was found between the URICA scores and EDE-Q scores (Pearson’s *r* = −0.27, *p* = 0.023). A linear regression analysis was performed with the URICA scores as a predictor of variance of the EDE-Q scores. The URICA scores were found to explain 7.5% of the variance in the EDE-Q scores. In Group 2, 32 patient episodes were included in the analysis, as data was available for 35 patient episodes, and three outliers were excluded. A significant negative correlation of moderate strength was found in this group (Pearson’s *r* = −0.49, *p* = 0.003). A linear regression analysis was performed with the URICA scores as a predictor of variance of the EDE-Q scores. The URICA scores were found to explain 23.9% of the variance in the EDE-Q scores.

### Correlations between the ANSOCQ scores and severity

The ANSOCQ and EDE-Q scores were correlated in Groups 1 and 2. In Group 1, 22 patient episodes were analysed, and no outliers were excluded. A significant negative correlation of moderate strength was observed between the ANSOCQ and EDE-Q scores (Pearson’s *r* = −0.45, *p* = 0.036). The ANSOCQ scores accounted for 20.2% of the variance observed in the EDE-Q scores. In group 2, data was available for 15 patient episodes, and no outliers were excluded. A negative correlation of moderate strength was observed between the ANSOCQ and EDE-Q scores, although this was not significant (*r* = 0.395, *p* = 0.145).

## Discussion

Several key findings were identified in this study: the URICA has limited value as a predictive tool for weight gain in AN; the ANSOCQ has a moderately positive predictive value for weight gain in AN; the URICA and ANSOCQ scores were significantly correlated with each other; the URICA scores correlated significantly with symptom severity, measured by the EDE-Q in Groups 1 and 2; the ANSOCQ scores also correlated significantly with symptom severity in Group 1, and whilst they were not significantly correlated with the EDE-Q scores in Group 2, the correlation was of a moderate effect size.

This study found that the URICA score is a poor predictor of weight gain in patients with AN following outpatient treatment at BETRS. This calculation was taken from Group 2 only, which included patients who had all received treatment at BETRS. A sample size of 46 patients was analysed. There was found to be a very weak correlation strength, and no statistical significance for this result. This finding is fitting with the authors’ initial hypothesis. As discussed earlier, the URICA scale has not been validated or designed for use in an eating disorders population. Rieger et al., [22] posit that the URICA scale, is therefore likely to overestimate the readiness to change in an eating disorder population, making it a poor tool for predicting prognosis [22].

The ANSOCQ on the other hand was found to have moderate and significant predictive value for weight gain which is fitting with prior research [22] and the predictions of the authors.

The URICA and ANSOCQ scores were significantly correlated, with moderate strength in Groups 1 and 2. The URICA scores were found to explain 22 and 21% of variance in the ANSOCQ scores respectively in Groups 1 and 2.

A question is raised around why the ANSOCQ and URICA scores are correlated, but only the ANSOCQ scores were significantly predictive of weight gain. This can potentially be explained by a closer inspection of the two questionnaires. There are similarities between the two scales. The ANSOCQ was initially developed from a range of existing instruments, including the URICA scale. It is evident when reading the two scales that there are similarities in structure and wording. Both scales are categorical in nature, and structured around the Prochaska DiClemente stages of behavioural change [13]. This may explain the correlation between the two scales.

However, the ANSOCQ differs from the URICA in that the questions are specific and focused on AN-specific symptoms. Whereas, the questions posed in the URICA scale are less focused and potentially more open to interpretation by the patient. Hence, the URICA scale may be less likely than the ANSOCQ to capture stage of change with respect to AN. This may explain why only the ANSOCQ scores were predictive of weight gain following treatment.

Another key finding of this study was that the URICA scores and ANSOCQ were predictive of symptom severity. The URICA scores were significantly predictive of symptom severity in Groups 1 and 2. A moderate correlation was also observed between the ANSOCQ and EDE-Q scores in Groups 1 and 2, however this result was only statistically significant in Group 1. This result can likely be explained by insufficient available data in Group 2 (*n* = 15), compared to Group 1 (*n* = 22). With higher available data in Group 2, a statistically significant outcome may have been observed.

It is important to highlight that the patient sample considered in this study is a clinical sample and not a research sample. A strength of this patient sample is that all patients recruited into this study were actual patients treated at a public mental health service, and referred by primary and secondary care providers for clinical reasons, rather than recruited specifically for research purposes. They are therefore a representative clinical sample. The cohort sample size was strong, including 119 patients who were assessed at BETRS (Group 1), and sub-group of 63 patients who were assessed and received treatment (Group 2). Another strength of this study was the use of uniform assessment using standardised instruments.

Limited sample size for several of the analyses was a weakness of the study, although it should be noted that statistically significant outcomes were still achieved for most analyses. Limitations of this sample included significant gaps in data due to incomplete compliance with assessment packages in those who consented to participate.

Patient numbers were also limited by lack of consent, with 35.74% of eligible participants failing to provide consent for the use of their data in this study. It is important to note that the lack of consent was not specific to this study but referred to the general consent sought for use of data collected for the larger BETRS data base and subsequent research use rather than clinical use. Furthermore, lack of consent for a majority of these participants coincided with lapses in administrative and research staffing in BETRS, and is therefore not necessarily linked with a refusal to participate in research. No inference can be drawn regarding the non-consenters motivation for change and the impact their exclusion from this study might have.

Another limitation in the data available was the limited outcome measures recorded on discharge. This study used weight gain as an indicator of success in patients with AN treated at BETRS, however other measures of success would potentially have enhanced this study further such as the EDE-Q on discharge.

It is important to revisit the question of whether the TTM is of value in understanding and treating AN, in light of the contributions of this study. As mentioned above, the TTM has been the main conceptual model used in understanding the lack of motivation in patients with AN yet the evidence for the use of this model in AN has limitations. Existing data is limited, and the few studies investigating the TTM in AN outcomes lack methodological consistency, including how the stage of change is defined and measured [9].

This study found that the URICA Scale which measures readiness to change, had no significant predictive value for treatment outcomes in terms of weight gain. This may be because the URICA Scale is not appropriate for measuring stage of change in patients with AN. However, the ANSOCQ which is specific for stage of change in AN had a moderate correlation with and accounted for one-third of variance in weight gain. The overall findings of this study therefore support existing literature suggesting that stage of change and the TTM have an important prognostic value in the assessment of AN.

These findings have implications for clinical practice and further research. This study found that the URICA Scale is of limited use for predicting treatment outcomes or measuring stage of change in adults with AN. Utilisation of self-report scales is a resource intensive practice. As such, assessing stage of change, using the URICA may be of limited utility in clinical practice in the assessment and treatment of AN. The ANSOCQ however was found to have significant predictive value in predicting weight gain in adults with AN, and assessing stage of change. This is fitting with prior research, which supports the prognostic value of the TTM in an AN population. . This study demonstrates that the ANSOCQ is a more appropriate tool for assessing stage of change and should be used in further research in AN in favour of the URICA. The ANSOCQ also has value clinically in predicting treatment outcomes. This is highly important in providing education to patients and families about the likely outcomes of illness and treatment planning.

In terms of direction for future research, there are several gaps in the current literature. There is a deficit of research in adults with AN with a majority focussing on adolescents [10, 13–15]. There is also a deficit of research in outpatient settings, with a majority of research focused on hospitalised patients. Most patients with AN are treated in outpatient settings, [26] which would suggest that this is a key population for future research.

## Conclusions

This study has filled an identified gap in research. To the author’s knowledge it is the only study evaluating stage of change and the TTM in an adult outpatient population with AN. This study used a true clinical rather than a research sample and, as such, the conclusions of this study should be generalizable to clinical practice.

The findings of this study suggest that the URICA is a poor tool for evaluating stage of change, and is therefore of limited clinical value. The ANSOCQ is a more accurate and appropriate tool for assessing stage of change, and predicting treatment outcomes in outpatients with AN in an outpatient population, and may helpfully inform clinical practice in terms of directing treatment and informing prognosis. This finding is in keeping with prior research which has focused on inpatients with AN [22, 23].

## Abbreviations

AN: Anorexia Nervosa
ANSOCQ: Anorexia Nervosa Stage of Change Questionnaire
BETRS: Body Image Eating Disorders Treatment and Recovery Service
BMI: Body Mass Index
EDE-Q: Eating Disorder Examination Questionnaire
SD: Standard deviations
URICA: University of Rhode Island Change Assessment Scale
