# Correction: An evaluation of the predictive validity of the URICA and ANSOCQ scales for weight gain in adults with AN in an outpatient eating disorders program: a prospective cohort study

**DOI:** 10.1186/s40337-023-00923-8

**Published:** 2023-11-13

**Authors:** Jessica Green, Andrea Phillipou, David Castle, Leonardo Cistullo, Richard Newton

**Affiliations:** 1 Austin Hospital, Heidelberg, Australia; 2 Swinburne University, Hawthorn, Australia; 3 St Vincent’s Health, East Melbourne, Australia; 4 University of Melbourne, Parkville, Australia; 5 Monash University, Clayton, Australia; 6 Peninsula Health, Frankston, Australia

**Correction: Journal of Eating Disorders (2017) 5:50** **https://doi.org/10.1186/s40337-017-0180-0**

Following publication of the original article [1], the authors identified an error in the author’s name of Andrea Phillipou.

The incorrect author’s name is Andrea Philipou. The correct author’s name is Andrea Phillipou. The author group has been updated above and the original article [1] has been corrected.
